# Changyanning regulates gut microbiota and metabolism to ameliorate intestinal injury induced by ETEC K88

**DOI:** 10.3389/fmicb.2023.1098818

**Published:** 2023-01-26

**Authors:** Pei Guo, Zongke Wang, Xiaojing Lv, Xin Wang, Jiaying Yu, Xuelei Tian, Hu Shan, Zhihua Qin

**Affiliations:** ^1^College of Veterinary Medicine, Qingdao Agricultural University, Qingdao, Shandong, China; ^2^QingDao Xnoba Biological Technology Co., Ltd., Qingdao, Shandong, China

**Keywords:** Changyanning, diarrhea, gut microbiota, metabolomics, ETEC K88

## Abstract

*Enterotoxigenic Escherichia coli* (ETEC) is a common pathogen of swine colibacillosis, which can causing a variety of diseases initiate serious economic losses to the animal husbandry industry. The traditional Chinese medicine Changyanning (CYN) often used for diarrhea caused by the accumulation of damp heat in the gastrointestinal tract, has anti-bacterial, anti-inflammatory and anti-oxidation effects. This study investigated the effect of CYN on gut microbiota and metabolism in mice infected with ETEC K88. A total of 60 Kunming mices were divided into Control group, ETEC K88 group, CYN.L group (2.5 g/kg), CYN.M group (5 g/kg), CYN.H group (10 g/kg) and BTW group (10 g/kg), determined clinical symptoms, intestinal morphology, inflammatory responses, gut microbiota as well as serum metabolites. CYN administration elevated ETEC K88-induced body weight loss, ameliorated duodenum, ilem, colon pathological injury, and reduced the increase of spleen index caused by ETEC. CYN also reduced the levels of pro-inflammatory cytokines (IL-6, TNE-α) in the serum. 16s rRNA gene sequencing results showed that CYN increased the abundance of beneficial bacteria *Lactobacillus* but decreased the abundance of pathogenic bacteria *Escherichia* in the feces of mice. Moreover, CYN participates in amino acid biosynthesis and metabolism in the process of serum metabolism to regulates ameliorate intestinal injury induced by ETEC K88. In conclusion, CYN regulates gut microbiota and metabolism to ameliorate intestinal injury induced by ETEC K88.

## 1. Introduction

Bacterial infection is an important factor endangering the healthy development of animal husbandry. E.coli is the important pathogen causing intestinal diseases in humans and animals (Bin et al., 2018). Among them, *Enterotoxigenic Escherichia coli* (ETEC) is a common pathogen of swine colibacillosis, which can easily induce enteritis and enterotoxemia, causing diseases such as piglet yellow diarrhea, piglet pullorum, and piglet edema disease (Kim et al., 2022). The main virulence factors of ETEC K88 are adhesins and enterotoxins (Dubreuil, 2021). ETECs can colonize the intestinal epithelium through virulence factors such as adhesins, thus leading to increased intestinal permeability and an inflammatory response (Wan et al., 2018; Li H. et al., 2021). At the same time, it will also lead to stunted growth and death of piglets, decrease in feed utilization rate, and cause serious economic losses to the animal husbandry industry (Laird et al., 2021). Antibiotics and heavy metal compounds, such as zinc oxide and copper sulfate, are widely used in pig industry to treat ETEC infection. However, the problem of antibiotic resistant bacteria and heavy metal pollution has aroused great concern. Therefore, it is very important for livestock production to find alternative resistance and treatment methods for ETEC.

At present, the use of traditional Chinese medicine is spreading around the world. With its characteristics of multi components and multi targets, it has unique and good clinical effects on diseases (Li Y. et al., 2021).In recent years, researchers have found that traditional Chinese medicine can play an important role in regulating intestinal microbiota to treat diarrhea caused by ETEC. For example, HNa could alleviate ETEC K88-induced intestinal dysfunction through restoring intestinal barrier integrity, modulating gut microbiota, and metabolites (Wang et al., 2022). Micro-encapsulated (protected) organic acids (OA) and essential oils (EO) combination, protect gut barrier by increasing tight junction protein expression and increase Lactobacillus and Bacilli abundances after ETEC F4 (K88+) challenge (Xu et al., 2020). Ma et al. investigated the role of tea polyphenols in ETEC K88 infection using a mouse model. Pretreating with tea polyphenols attenuated the symptoms induced by ETEC K88 (Ma T. et al., 2021).

The traditional Chinese medicine Changyanning (CYN) is composed of five kinds of Chinese medicinal herbs: Dijincao (*Euphorbiae humifusae herba*), Huang Mao Er grass (*Hedyotis chrysotricha herba*), Camphor tree root (*Cinnamomum camphora root*), Elsholtziaciliata (*Moslae herba*), and Maple leaves (*Liquidambar formosana leaves*). It has the effects of clearing heat and detoxifying, and moistening the intestines, so it is often used for diarrhea caused by the accumulation of damp heat in the gastrointestinal tract. In addition, modern pharmacological studies have shown that CYN has antibacterial, anti-inflammatory and relaxant effects on intestinal smooth muscle (Hu and Yang, 2022). The research confirmed that Changyanning granules have a significant effect on the treatment of ulcerative colitis in mice caused by DSS, can alleviate intestinal inflammation and protect intestinal health (Hu and Yang, 2022). A recent study showed that the CYN formula is an effective anti colitis therapy, the underlying pharmacological mechanisms of which involve anti-inflammation and anti-oxidation, as well as the re-establishment of a healthy microbiota community (Yu et al., 2022).

At present, Changyanning has been used in DSS models for research, whether it also has a role in the mice model infected by ETEC K88? We have carried out research around this issue to verify that CYN in the treatment of acute diarrhea caused by ETEC K88, and provide ideas for veterinary clinical development of new veterinary drugs. Therefore, the objective of this study is to investigate the therapeutic effects and the mechanism of action for the oral administration of CYN on ETEC K88-infected mice by determining intestinal morphology, intestinal microbiota as well as metabolites.

## 2. Materials and methods

### 2.1. Materials

CYN medicinal herbs purchased from Chengyang People’s Hospital and Tongfang Hospital of Qingdao City, Shandong Province, Dijincao, Huang Mao Er grass, Elsholtziaciliata and Maple leaves in 4:5:4:2:2 proportion. The procedure is as follows: extraction with three times of water is carried out, then heated and refluxed twice, filtered using a vacuum pump, combined the filtrate twice and fixed, and concentrated the filtrate to a final volume of 1.0 g/mL (calculated according to the crude drug amount).

Baitouweng oral solution was collected in Commission of Chinese Veterinary Pharmacopoeia (2020) and purchased from Hefei Qianfang Dynamic Protection Technology Co., Ltd. It has the functions of clearing heat and detoxifying, cooling blood and stopping dysentery, is often used in traditional Chinese medicine for the treatment of *E. coli* diarrhea in pigs.

The *E. coli* strain ETEC K88 was maintained in our laboratory. ETEC K88 was cultured in Luria-Bertani broth at 37°C for 12 h and centrifugated at 3,000 × g for 10 min at 4°C after being incubated on the shaker. Cells were washed with sterilized phosphate buffer saline (PBS) three times. Finally, ETEC K88 concentration was adjusted to 2 × 10^10^ colony-forming unit (CFU) mL^−1^ in PBS.

### 2.2. Animals, experimental design, and sample collection

Male-specific pathogen-free (SPF) Kunming mice weighing 20.0 ± 2.0 g (6–8 weeks) were purchased from Jinan Pengyue Experimental Animal Breeding Co., Ltd., Jinan, China. All mices were raised in an animal room free of specific pathogens in Qingdao Agricultural University, and were given free access to water and fed a commercial diet. The temperature was controlled at 24 ± 2°C, the relative humidity was 60 ± 5% with a 12 h light–dark cycle. The mices were acclimatized to laboratory conditions for 1 week prior to being subjected to the experiments. All animal experiments were performed in accordance with the Guidelines of Animal Ethics Committee of Qingdao Agricultural University.

After 1 week of adaptation, 60 mices were divided into control group, ETEC K88 group, CYN.L group (2.5 g/kg), CYN.M group (5 g/kg), CYN.H group (10 g/kg), and BTW group (10 g/kg). From day 1, all groups except control group were intraperitoneally injected with 0.2 mL ETEC K88 solution at a concentration of 2 × 10^8^ cfu/mL. From the second day of the experiment, the control group and ETEC K88 group were given normal saline by oral gavage, and the CYN groups and BTW group were given corresponding drugs for 7 days, respectively.

During the experiment, the stools, coats and mental states of the mice were observed, and the body weights of each group of mice were measured and recorded at a fixed time point every day. After 7 days of gavage, the mice were fasted for 24 h and killed. Blood was collected from the orbital venous plexus, left at room temperature for 1 h and centrifuged for 15 min (167.7 × g); the serum was then collected and stored at-80°C. Subsequently, the mice were sacrificed by cervical dislocation, then the spleens were weighed immediately to calculate the spleen index. Spleen index (%) = spleen weight (g)/body weight (g) × 100%. The isolated colon were cut to 1-cm lengths and fixed in 4% paraformaldehyde for HE staining and histological examination. Fresh feces were collected and stored at-80°C, transported to SangonBioTech for 16S rRNA high-throughput sequencing.

### 2.3. Pathological analysis

The duodenum, ilem, colon tissues tissue samples were fixed in 4% paraformaldehyde fix solution. The histologic section processed as previously described (Qing et al., 2017). Subsequently, the fixed intestinal tissues are processed, trimmed, and embedded in paraffin. Sections with 5 μm thickness were stained with hematoxylin and eosin (HE). An optical microscope was used to observe morpho-logical changes.

### 2.4. Enzyme-linked immunosorbent assay

Mice blood was collected in centrifuge tubes, left standing at room temperature for 1 h, and then centrifuged at 1509.3 × g for 10 min after stratification. The supernatant was centrifuged at 24148.8 × g for 10 min at 4°C, and the new supernatant was dispensed into new centrifuge tubes. TNF-α, IL-6, IL-10 kits were purchased from Nanjing Jiancheng Bioengineering Institute, and the experiments were carried out according to the instructions.

### 2.5. 16S rRNA analysis of fecal samples

Fecal samples were collected and immediately placed in liquid nitrogen and stored at –80°C. The 16S rRNA analysis of the fecal samples was performed at Sangon Biotech (Shanghai) Co., Ltd., Shanghai, China. The genomic DNA of fecal samples was extracted by an E.Z.N.A.® soil DNA Kit (Omega Bio-Tek, Norcross, GA, United States) following the manufacturer’s instruction and detected by 1% agarose gel electrophoresis. The V3-V4 hypervariable regions of the microbial 16S rRNA gene were amplified by universal primers 341F (5′ -CCTACGGGNGGCWGCAG-3′) and 785R (5′ -GACTACHVGGGTATCTAATCC-3′). The PCR amplification system was 30 μL, and all products were purified by Hieff NGS™ DNA Selection Beads (Yeasen, 10105ES03, China), and quantified by Qubit® 4.0 Green double-stranded DNA assay. The purified PCR products were used to construct a sequencing library according to the specifications of Illumina TruSeq (Illumina, United States), and the quality detection and fluorescence quantification of the initial library were performed.

Using Usearch software (version 11.0.667) carried out high-quality sequence clustering to eliminate chimeras sequences and obtain Operational Taxonomic Units (OTUs) with 97% similarity. Before diversity analysis, rarefaction curves were generated to assess the sequencing depth of each sample. According to the measured effective data, alpha diversity and beta diversity were analyzed successively. Multiple indicators such as Shannon, ACE, Chao1, and Good’s coverage were calculated, and the similarities and differences of the gut microbial communities of each group were observed. To estimate the diversity of the microbial community of the sample, we calculated the within sample diversity by *T*-test for two groups and multiple group comparisons were made using ANOVA test. The false discovery rate corrected for *p* < 0.05 was considered significant for differences.

### 2.6. Serum metabolomics

Serum metabonomics analysis of control group, ETEC K88 group, CYN.M and BTW group were conducted by Biotree Biotech (Shanghai, China). A total of 100 μL of serum sample was added with 300 μL of solution (methanol, containing isotopically-labeled internal standard mixture). The samples were vortexed for 30 s and centrifuged at 13,800(×g), *R* = 8.6 cm for 15 min at 4°C. The supernatant was transferred to a 200 μL vial for analysis.

LC–MS/MS analyses were performed using an UHPLC system (Vanquish, Thermo Fisher Scientific) with a UPLC HSS T3 column (2.1 mm × 100 mm, 1.8 μm) coupled to Orbitrap Exploris 120 mass spectrometer (Orbitrap MS, Thermo). The mobile phase consisted of 5 mmol/L ammonium acetate and 5 mmol/L acetic acid in water (A) and acetonitrile (B). The auto-sampler temperature was 4°C, and the injection volume was 2 μL. The OrbitrapExploris 120 mass spectrometer was used for its ability to acquire MS/MS spectra on information-dependent acquisition (IDA) mode in the control of the acquisition software (Xcalibur, Thermo). In this mode, the acquisition software continuously evaluates the full scan MS spectrum. The ESI source conditions were set as following: sheath gas flow rate as 50 Arb, Aux gas flow rate as 15 Arb, capillary temperature 320°C, full MS resolution as 60,000, MS/MS resolution as 15,000 collision energy as 10/30/60 in NCE mode, spray Voltage as 3.8 kV (positive) or ˗3.4 kV (negative). The raw data were converted to the mzXML format using Proteo Wizard and processed with an inhouse program, which was developed using R and based on XCMS, for peak detection, extraction, alignment, and integration.

The internal standard normalization method was employed in this data analysis. The final dataset containing the information of peak number, sample name, and normalized peak area was imported to SIMCA16.0.2 software package (Sartorius Stedim Data Analytics AB, Umea, Sweden) for multivariate analysis. In order to visualize group separation and find significantly changed metabolites, supervised orthogonal projections to latent structures-discriminate analysis (OPLS-DA). Furthermore, the value of variable importance in the projection (VIP) of the first principal component in OPLS-DA analysis was obtained. The metabolites with VIP > 1 and *p* < 0.05 (student *t*-test) were considered as significantly changed metabolites.

### 2.7. Statistical analysis

The data were analyzed using the SPSS 21.0 statistical software, and the experimental results were all expressed as the mean ± standard deviation (SD). The statistical processing of the data used one-way analysis of variance (ANOVA), and the difference was tested as the means between multiple groups. A value of *p* < 0.05 was regarded to be statistically significantly different.

## 3. Results

### 3.1. Effect of CYN on clinical symptoms of ETEC K88-induced mice

**Figure 1:**
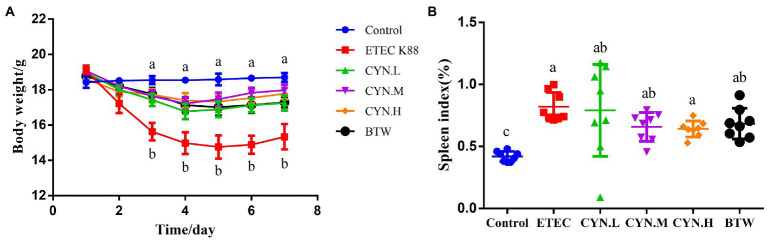
The effect of CYN on body weight and spleen index of mices infected by ETEC K88. **(A)** The body weight of each group. **(B)** Spleen index of mice. All of the data are expressed as the mean ± SEM. Different superscript lowercase letters within each group indicate significantly different (*p* < 0.05).

No mortality was observed in the treatment groups during the experimental period. After 4 h of ETEC K88 treatment, it was found that the mental state of mice was slightly poorer, but there was no diarrhea. After 8 h the mice began to show signs of illness, with messy coats and yellow water stools in each group, except the control group. The weight changes of mice in each group are shown in Figure 1A. Prior to intraperitoneal ETEC K88 injection, there was no significant difference in the weight of mice in each group (*p* > 0.05). ETEC K88 group showed body weight loss in comparison with the control group (*p* < 0.05). However, CYN groups and BTW group showed weight loss trend during 1–3 days after infection and the weight gain was improved and had no difference with the control group (*p* > 0.05). As shown in Figure 1B, compared with the control group, the value for spleen index of the mice injected the ETEC K88 groups increased significantly (*p* < 0.05), Dose-dependent reductions in the index values for various organs were observed in the CYN groups, but the changes were not significant.

### 3.2. Effect of CYN on intestinal injury and inflammation of ETEC K88-induced mice

**Figure 2:**
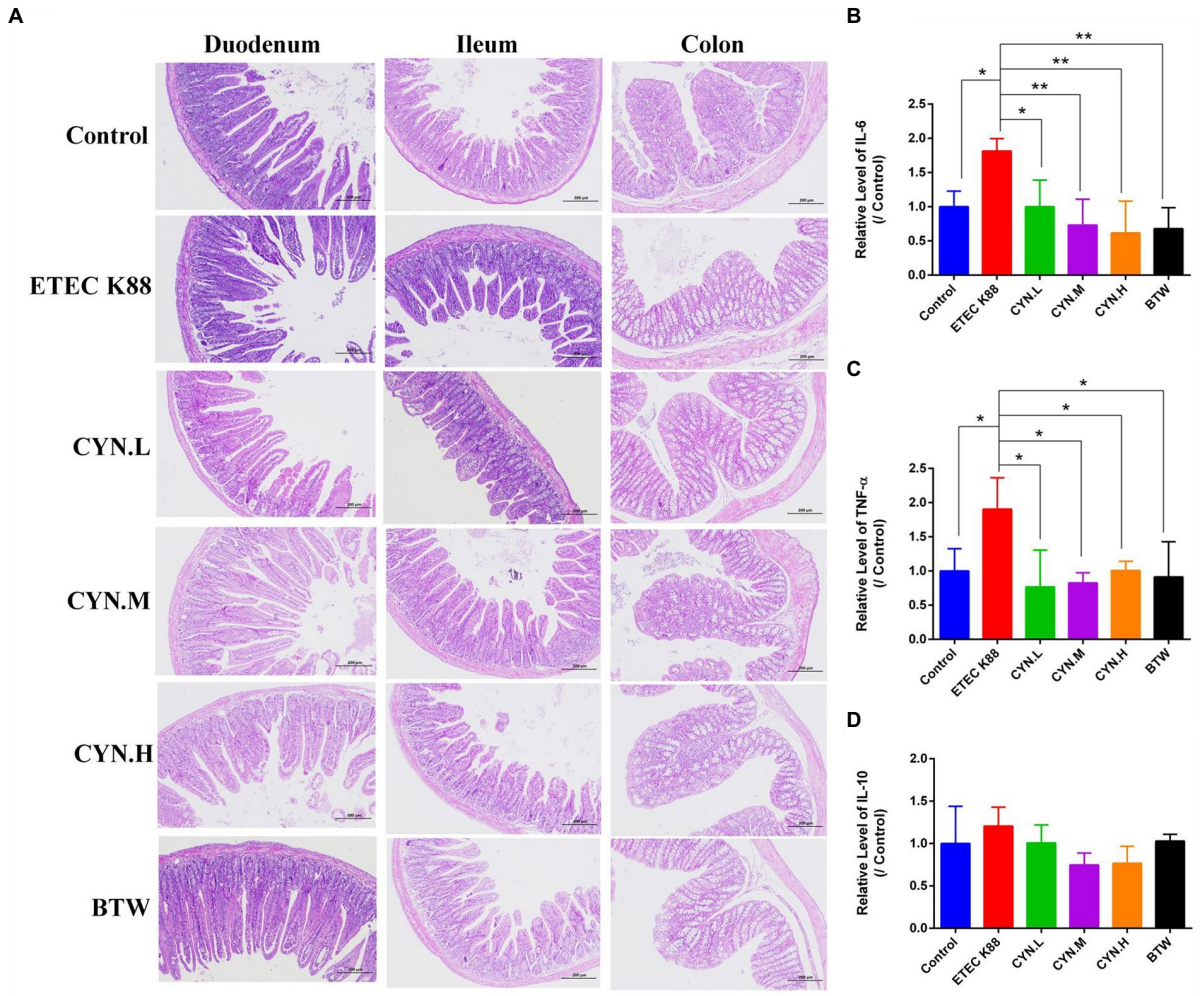
The effect of CYN on intestinal injury and inflammation of mices infected by ETEC K88. **(A)** The HE staining of the duodenum, ilem, colon tissues. **(B)** The changes of IL-6 content in each group. **(C)** The changes of TNF-α content in each group. **(D)** The changes of IL-10 content in each group. *p < 0.05, **p < 0.01, *n* = 3. The H&E stained sections presented at × 100 original magnification.

The results of intestinal histopathology showed that the epithelium of duodenum, ilem, colon mucosa in the control group was intact, arranged closely and orderly, the villi were arranged in a regular and orderly manner. In ETEC K88 group, goblet cells were enlarged, mucus appeared, villi became shorter, flattened and vacuolated. After treatment with CYN, intestinal tissue recovered to a certain extent, goblet cell swelling reduced, and intestinal villi arranged in a regular order (Figure 2A). This suggested that CYN could ameliorate intestinal injury induced by ETEC K88.

To study whether CYN can reduce the production of inflammatory factors in mice stimulated by ETEC K88, the levels of IL-6, TNF-α, IL-10 in mice serum were determined using Enzyme-linked immunosorbent assay (ELISA), and the anti-inflammatory activity of CYN was studied. As shown in Figures 2B,C, IL-6 and TNF-α secretion was significantly increased in the ETEC K88 group compared with the control group (*p* < 0.05). After treatment with CYN and BTW, the levels of IL-6 and TNF-α in CYN.L, CYN.M, CYN.H, and BTW groups were significantly lower than those in ETEC K88 group (*p* < 0.05). As shown in Figure 2D, the level of IL-10 in serum did not change significantly in each group. The results show that ETEC K88 can induce inflammation in mice and that CYN can significantly reduce the levels of IL-6 and TNF-α in mice serum and alleviate the inflammatory response in mice.

### 3.3. Effect of CYN on gut microbiota of ETEC K88-induced mice

**Figure 3:**
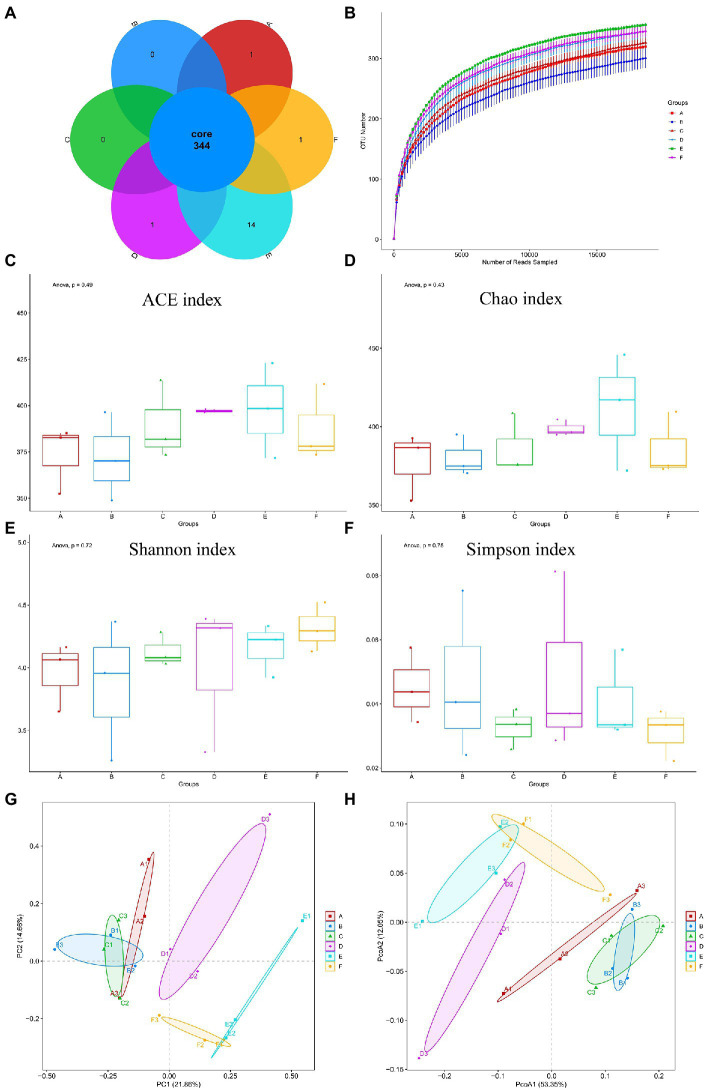
Number of bacterial OTUs and diversity index statistics in mice feces in six groups. **(A)** Venn diagram, where areas of overlap indicate the numbers of OTUs shared among the overlapping groups. **(B)** Alpha index spar curve, determine whether the amount of sequencing data is enough according to the curve flat. **(C)** ACE index. **(D)** Chao1 index. **(E)** Shannon index. **(F)** Simpson index. **(G)** PCA based on OTU level. **(H)** PCoA based on OTU level. Each point represents a sample and the distance between points is used to map the size of the difference in the structural composition of the gut flora between the two samples. In the picture, A represents Control group, B represents ETEC K88 group, C represents CYN. L group, D represents CYN. M group, E represents CYN. H group, and F represents BTW group.

Intestinal damage is accompanied by changes in the intestinal flora, which can be detected by 16S-rRNA. According to venn diagrams (Figure 3A) showed the OTUs of each group, both the same strain and different strains among different groups, including 344 strains of the same strains. Including 344 identical OTUs in different groups. As shown in Figure 3B, by drawing a sparse curve of each group at the OTU level, it was found that all curves tended to stabilize, indicating that a large number of microorganisms present in the samples were captured at this sequencing level, and that the result is credible. We computed alpha and beta diversity indexes that could reflect gut microbial diversity. We analyzed the number of species in each group by alpha diversity analysis on the composition of flora in each group to predict the abundance and diversity of gut microbiota. Among them, Chao, Ace index are used to calculate the difference of bacterial species abundance of intestinal flora between groups, and Shannon, Simpson index are used to calculate the difference of bacterial species diversity of gut microbiota. Comparative alpha diversity analysis of microbial diversity indices showed that there was no significant difference in the Chao1 (Figure 3C), ACE (Figure 3D), Simpson (Figure 3E), and Shannon (Figure 3F) indices between control and experimental groups regardless of the treatment dose, showed that CYN administration had no significant effect on the intestinal microbial diversity and abundance of mice infected with ETEC K88.

Principal component analysis (PCA) and principal coordinate analysis (PCoA) analysis were used to compare the similarity of the microbial community structures in different groups, were applied to dissect the gut microbial beta-diversity. It can be seen from the PCA and PCoA score chart although the alpha diversity of intestinal flora was not significantly different among the six groups, the classification composition of microbiome in ETEC K88 group was different from that in control group and CYN groups (Figures 3G,H).

**Figure 4:**
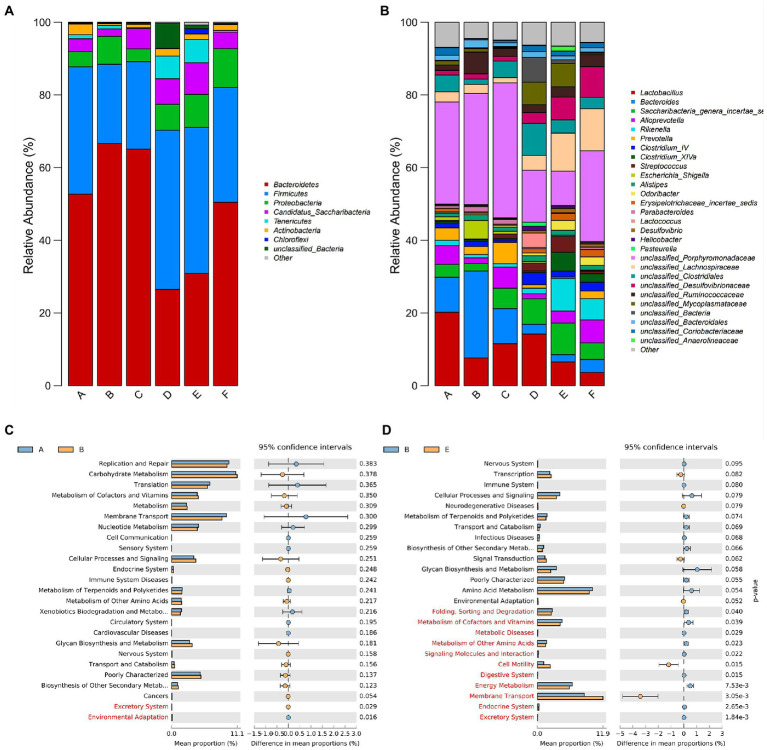
Microbial community composition **(A)** phylum levels. **(B)** Genus levels. The different Metabolic pathways prediction **(C)** Control vs. ETEC K88. **(D)** ETEC K88 vs. CYN.H. In the picture, A represents Control group, B represents ETEC K88 group, C represents CYN. L group, D represents CYN. M group, E represents CYN. H group, and F represents BTW group.

In addition to richness and diversity, changes in gut microbiota can also be reflected in the category and structure of the microbiota. Combined with Heatmap and microbiota structure bar chart analysis, it could be seen that the composition types of gut microbiota in each group of samples were similar, but the proportions were different. At the phylum level (Figure 4A), the gut microbiota of mice in each group mainly included *Bacteroidetes*, *Firmicutes*, *Proteobacteria*, *Saccharibacteria*, *Tenericutes*, *Actinobacteria*, *Chloroflexi* etc. The proportion of *Bacteroidetes* in the control group, ETEC K88 group, CYN.L group, CYN.M group, CYN.H group, and BTW group were 52.697, 66.624, 65.075, 26.467, 30.930, and 50.494%. *Firmicutes* accounted for 35.020, 21.815, 24.085, 43.831, 40.132, and 31.542%. We found that ETEC K88 infection decreased the relative abundance of *Firmicutes* and *Firmicutes/Bacteroidetes*, and increased the relative abundance of *Proteobacteria* (*p* < 0.05) whereas CYN treatment reversed that.

At the genus level (Figure 4B), it mainly included *Lactobacillus*, *Bacteroides*, *Rikenella*, *and Parabacteroides*. In the control group, ETEC K88 group, CYN.L group, CYN.M group, CYN.H group, and BTW group, the proportion of *Bacteroides* was 9.634, 23.937, 9.649, 2.675, 2.030, and 3.565%. The results showed that the relative abundance of *Bacteroides* in CYN.M, CYN.H, and BTW groups was significantly higher than that in ETEC K88 group (*p* < 0.05). In addition, the proportion of *Lactobacillus* in the intestinal microbes of the ETEC K88 group of mice was significantly decreased, while the proportion of *Lactobacillus* increased after CYN intervention. It should be noted that the *Escherichia* level in ETEC K88 group was significantly higher than that in control group, and after CYN and BTW treatment the level returned to normal. It may acted by regulated the number of beneficial and harmful bacteria. Therefore, medium and high doses of CYN could regulate the structure of intestinal microbiota to normalize and improve the abnormal changes of intestinal microbiota caused by ETEC K88 in mice.

PICRUSt analysis was used to predict functional changes in the gut microbiota of ETEC K88-induced mice, and the predictions based on the KEGG database showed that metabolism related differential pathways between groups, where the top 25 metabolic pathways were listed by the *p*-value [Figure 4C (Control vs. ETEC K88 group) and Figure 4D (ETEC K88 vs. CYN group)]. The membrane transport, metabolism of other amino acids, metabolism of cofactors and vitamins, glycan biosynthesis and metabolism, metabolism of terpenoids and polyketides, cellular processes and signaling, transcription were the common pathways in the control and ETEC K88 groups and the ETEC K88 and CYN.H groups. Suggested that CYN may regulate the gut microbiota in ETEC K88 treatment by affecting the carbohydrate metabolism, lipid metabolism and amino acid metabolism, the clear mechanism needs further experimental verification.

### 3.4. Effect of CYN on serum metabolism of ETEC K88-induced mice

**Figure 5:**
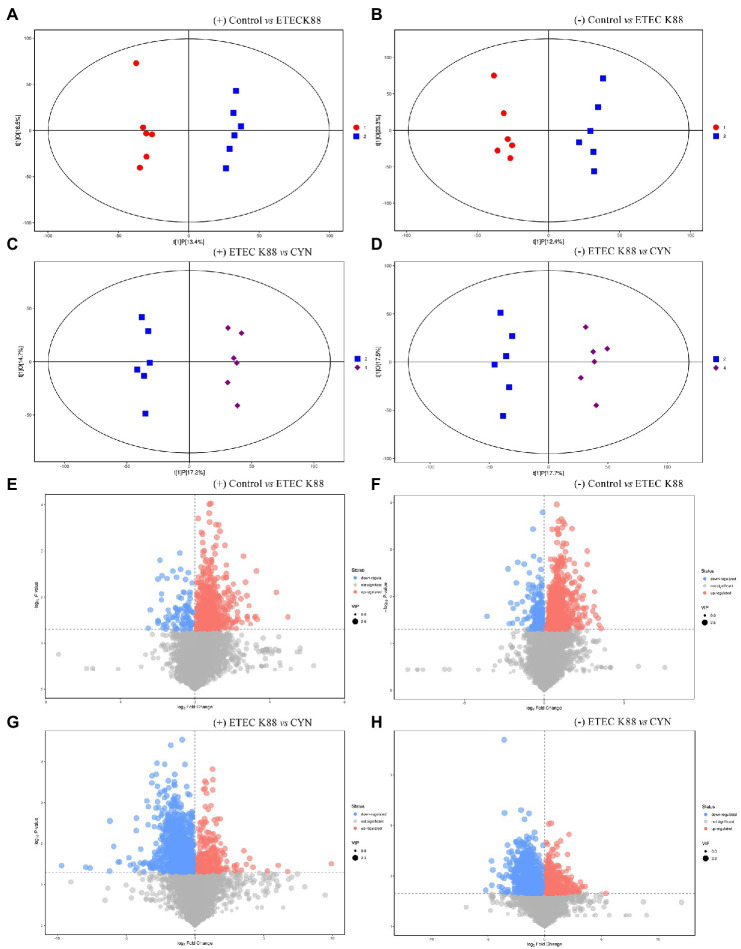
LC/MS metabolite principal component analysis and metabolite differences between groups. **(A,B)** OPLS-DA score plots based on the ESI (+) and (−) mode (Control vs. ETEC K88). **(C,D)** OPLS-DA score plots based on the ESI (+) and (−) mode (ETEC K88 vs. CYN). **(E,F)** Volcano plot based on the ESI (+) and (−) mode (Control vs. ETEC K88). **(G,H)** Volcano plot based on the ESI (+) and (−) mode (ETEC K88 vs. CYN). Metabolites with significantly up-regulated expression were in red, down-regulated expression were in blue, non-significant expression were in gray. In the picture, 1 represents Control group, 2 represents ETEC K88 group, 4 represents CYN group.

Metabolomics has been shown to be a great tool to reveal information about the crosstalk between the host and gut microbiota (Org et al., 2017). Non-targeted metabolomic detection was performed by liquid chromatography-mass spectrometry (LC/MS). The data of principal component analysis showed that the control group and ETEC K88 group, no matter in the positive ion mode or the negative ion mode, the samples all had significant clustering and metabolic profiles on two different dimensions of the PC2 axis (Figures 5A,B). Similarly, the sample metabolic profiles of the ETEC K88 group and the CYN group were also located at different positions of the PC2 axis in the positive-ion mode and the negative-ion mode (Figures 5C,D). These results indicated that the metabolites of ETEC K88 group were different from those of control group and CYN group. Subsequently, the overall distribution of metabolite differences between groups was visualized in the form of a volcano plot. Each dot on the volcano map represented a metabolite, and the larger the scatter, the greater the VIP value. In both positive and negative ion modes, ETEC K88 group has significantly difference compared with control group and CYN group (Figures 5E–H).

**Figure 6:**
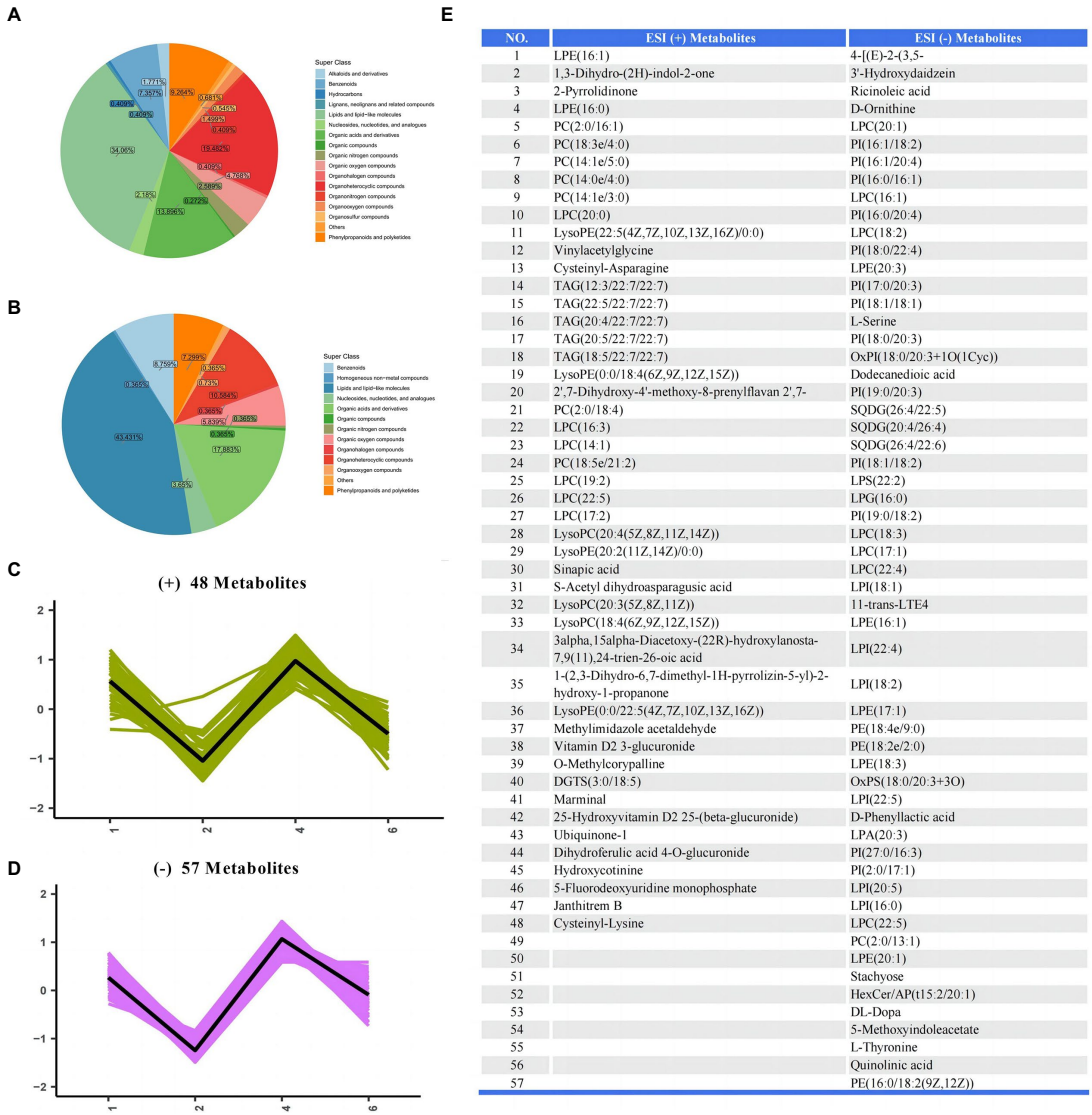
The differential metabolites of serum samples between groups. **(A,B)** Pie plot of metabolite classification based on the ESI (+) and (−) mode. **(C,D)** *K*-Means clustering analysis of different groups based on the ESI (+) and (−) mode. **(E)** The list of differential metabolites in serum.

The metabolites identified from all samples were classified according to the chemical classification information, as shown in Figures 6A,B. The major metabolites in the positive ion mode were lipids and lipid-like molecules (34.06%), organoheterocyclic compounds (19.482%), and organic acids and derivatives (13.896%). The major metabolites in the negative ion mode were lipids and lipid-like molecules (43.431%), organic acids and derivatives (17.883%), and organoheterocyclic compounds (10.584%). In order to study the change trend of the relative content of metabolites in different groups, the relative content of all different metabolites identified in the comparison of all groups according to the screening criteria was standardized by *z*-score, and then *K*-Means clustering analysis was carried out. The results showed that there have 57 metabolites in the positive ion mode and 48 metabolites in the negative ion mode among the groups were shown in Figures 6C–E.

**Figure 7:**
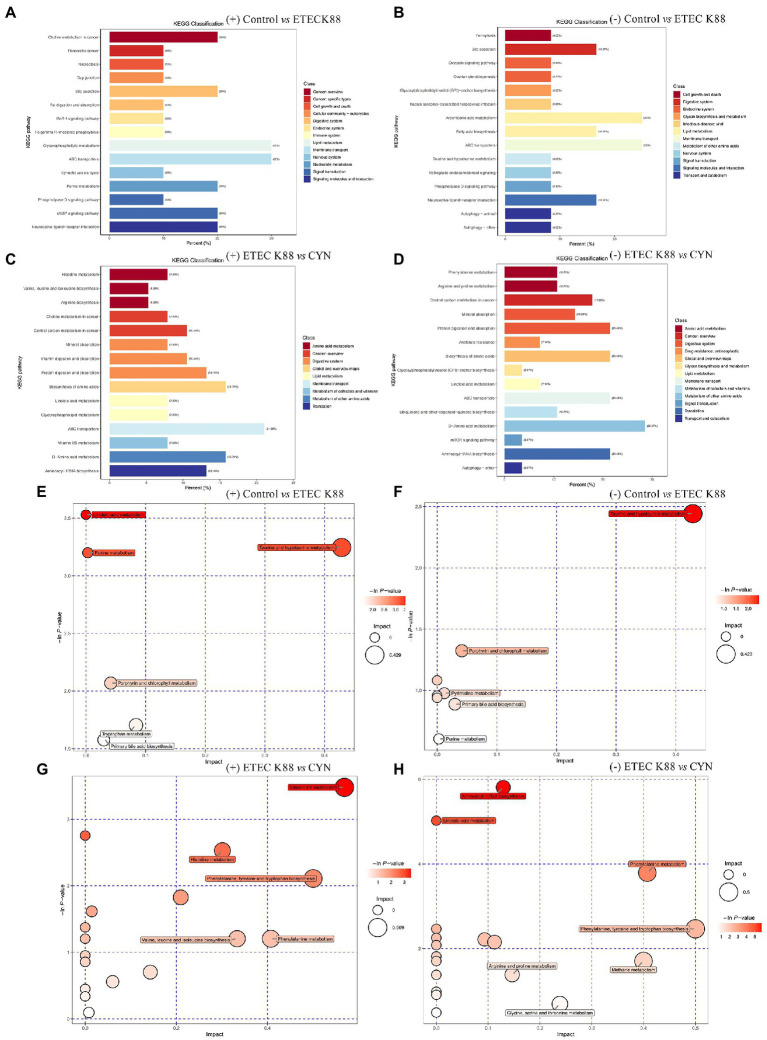
Major metabolic pathway analysis of serum samples between groups. **(A–D)** KEGG classification diagram on the ESI (+) and (−) mode. **(E–H)** Bubble plot of metabolic pathway based on the ESI (+) and (−) mode.

Then KEGG enrichment analysis was carried out, Compared with the control group, the main metabolic path ways altered in the ETEC K88 group included glycerophospholipid metabolism, ABC transporters, cAMP signaling pathway, purine metabolism, bile secretion, arachidonic acid metabolism, belongs to lipid metabolism, membrane transport, signal transduction, nucleotide metabolism, digestive system (Figures 7A,B). Compared with the ETEC K88 group, the metabolic pathways altered in the CYN group were D − Amino acid metabolism, protein digestion and absorption, biosynthesis of amino acids, ABC transporters, aminoacyl−tRNA biosynthesis belongs to metabolism of other amino acids, digestive system, membrane transport, translation (Figures 7C,D). In addition, to explore the pathways that CYN might affect, metabolites were further analyzed, and the results of metabolic pathway analysis were presented as bubble plots (Figures 7E–H). The pathways with influence >0.4 were screened as potential target pathways, and the pathway of aminoacyl−tRNA biosynthesis, linoleic acid metabolism, phenylalanine metabolism, phenylalanine, tyrosine and tryptophan biosynthesis, arginine and proline metabolism, methane metabolism, glycine, serine and threonine metabolism, vitamin B6 metabolism, histidine metabolism, valine, leucine, and isoleucine biosynthesis were obtained in ETEC K88 vs. CYN groups by comprehensive analysis of positive and negative ion patterns. In summary, CYN may play a role by participating in the biosynthesis and metabolism of amino acids.

## 4. Discussion

*Enterotoxigenic Escherichia coli* is a pathogenic *Escherichia coli* strain, which is one of the most important pathogens in weaned piglets (Hrala et al., 2021). Numerous studies elucidated that ETEC K88 infection could cause great economic loss in livestock industry due to poor growth performance and high mortality of animals (Qiu et al., 2017; Becker et al., 2020; Wang et al., 2020). In the present study, we investigated the protective potential of CYN against ETEC K88 infection in mice. The results revealed that the body weight of mice was decreased but the spleen index was increased after ETEC K88 infection, while CYN treatment reversed these changes. A previous study found serum cytokines could be used as biomarkers of mucosal inflammation as they have high correlations, which could be used for intestinal disease assessment (Ljuca et al., 2010). It was reported that pro-inflammatory factors such as IL-10, IL-6, and TNF-α are important indicators in response to ETEC K88 (Qiao et al., 2020). The present study found that ETEC K88 infection dramatically increased the concentrations of IL-6, and TNF-α in the serum. In the enteritis patient, TNF-α not only increase the infiltration of neutrophils, but also cause damage to the intestinal barrier (Cao et al., 2004). Wang et al. detected the inflammatory factors in the serum of mice infected with ETEC and found that ETEC could increase the levels of TNF-α and IL-6 (Wang et al., 2022), similar to our result. Collectively, these data suggest that CYN can inhibiting the production of pro-inflammatory cytokines of ETEC K88 infected mice.

The gut microbiota is recognized as an indispensable “Metabolic organ,” and there is increasing evidence that the effects of microbiome fluctuations on metabolism and physiological function may play an important role in the development of disease (Lu et al., 2014; Lin et al., 2018). In order to understand the mechanism of CYN regulate intestinal injury induced by ETEC K88, 16S-RNA method was used for further study. After analyzing the results of the study, it was found that the bacteria involved mainly included not only *Bacteroidetes, Firmicutes, Proteobacteria, Candidatus, Tenericutes* and *Actinobacteria*, but also *Lactobacillus, Bacteroides, Saccharibacteria*, and so on. And ETEC K88 increased the proportion of *Bacteroidetes* and decreased the proportion of *Firmicutes*, and CYN treatment alleviated the disrupted diversity of the gut microbiota. In a related study on diarrhea induced by ETEC, Yue et al. found that the number of *Firmicutes* in the intestinal flora of mice was significantly decreased after ETEC K88 infection (Yue et al., 2020). Wang et al. found that ETEC K88 infection decreased the relative abundance of *Firmicutes* and *Firmicutes/Bacteroidetes*, and increased the relative abundance of *Proteobacteria* (*p* < 0.05) (Wang et al., 2022). When Ma *et al* studied the preventive effect of DESP on diarrhea, they found that ETEC increased *Bacteroidetes* in the gut microbiota (Ma Y. et al., 2021). These studies confirmed that ETEC K88 caused intestinal microflora disorder, and this study also found that CYN can improve this condition.

It is well known that the changes in the number of intestinal pathogenic bacteria and probiotics can be used as an important indicator to maintain intestinal health (Dong et al., 2019). In fact *Lactobacillus* could improve intestinal health by inhibiting colonization of pathogens and enhancing mucosal immunity (Qiao et al., 2020). In this study, we also found that the number of *Escherichia* significantly increased and the number and ratio of *Lactobacillus* significantly decreased after ETEC K88 infection, suggesting that the intestinal flora of ETEC K88 mice was in disorder, thus reducing the intestinal resistance to pathogenic bacteria (Piewngam et al., 2018). In summary, the altered intestinal microbiota abundance of CYN administration may be related to its inhibition effects on pathogenic bacteria such as *Escherichia*. These studies further confirmed our results that CYN had beneficial effects on maintaining intestinal microbiota homeostasis. Thus, we speculate that CYN may alleviate intestinal dysfunction induced by ETEC K88 through maintaining intestinal homeostasis and increasing the abundance of intestinal beneficial bacteria.

Gut microbiota is widely involved in host metabolic activities, producing a variety of active metabolites that play important roles in maintaining a host’s intestinal barrier integrity and immune balance *via* providing nutrition to intestinal epithelial cells and activating various receptors directly or indirectly (Rowland et al., 2018). Gut microbiota and their major metabolites, short-chain fatty acids (SCFAs), are recognized as important players in gut homeostasis and metabolic disease occurrence. Notably, some increased bacterial genus such as *Prevotella* and *Odoribacter* in CYN group are potential producers of short-chain fatty acids (SCFAs) which are anaerobic gram-negative bacterium, can produce short chain fatty acids (SCFAs), modulate inflammatory responses, and exert an anti-inflammatory effect in patients with inflammatory bowel disease (Li W. et al., 2021). *Lactobacillus* is the only glycolytic probiotic that reduces intestinal permeability by producing various tryptophan catabolic products (indole, tryptamine, and tryptophol, among others), thereby maintaining the stability of the gut microbiome (Coras et al., 2020). In our study, CYN increased the reduction of Lactobacillus caused by ETEC K88 infection.

Since metabolome is a composite of metabolic activity of host and microbe (Wall et al., 2009), we further analyzed the serum metabolites. The analysis of serum metabolomics showed that CYN influenced amino acid biosynthesis and metabolism, meantime altered the concentrations of lipids and lipid-like molecules. Our findings were consistent with the study demonstrating that gut microbiota influences lipid metabolism and lipid levels in serum and tissues (Schoeler and Caesar, 2019). When investigating the protective effect of l-theanine on colitis, Zhang et al. analyzed the correlation between transcriptional profiles and lipid profiles and found that inflammation-related genes were almost significantly associated with differential lipid metabolites (Zhang et al., 2021). In addition, more and more studies show that amino acids also play an important role in preventing and treating intestinal inflammation (He et al., 2018). For example, aromatic amino acids (AAAs), including tryptophan, phenylalanine and tyrosine, reduce intestinal inflammation by activating calcium sensitive receptors (CaSR) in piglets (Liu et al., 2018). Moreover, it was also found that amino acids might regulate the composition of intestinal microbiota (Beaumont and Blachier, 2020), which coincided with the results of this study. Results indicated that CYN elevated the serum levels of phenylalanine metabolism, phenylalanine, tyrosine, and tryptophan biosynthesis pathway-related metabolites, phenylalanine, tyrosine, and tryptophan are important pathways of amino acid metabolism, and alterations in amino acid metabolism are closely correlated with inflammation (Mangal et al., 2021). In a previous study, speculated that the phenylalanine, tyrosine, and tryptophan biosynthesis and phenylalanine metabolism might be related to the regulation of the richness of *Lactobacillus*, *Romboutsia*, and *Bacteroides* in the gut (Cong et al., 2022), this rule was also found in this study.

Taken together, all of the above findings provide evidences that CYN may alleviate intestinal dysfunction induced by ETEC K88 through maintaining intestinal homeostasis and increasing the abundance of intestinal beneficial bacteria. CYN participates in amino acid biosynthesis and metabolism in the process of serum metabolism to regulates ameliorate intestinal injury induced by ETEC K88. These results provide a theoretical basis for the application of CYN in the treatment of intestinal diarrhea caused by ETEC K88, and provide ideas for veterinary clinical development of new veterinary drugs.

## Data availability statement

The raw data supporting the conclusions of this article will be made available by the authors, without undue reservation. The 16S rRNA sequencing data presented in the study are deposited in the NCBI repository, accession number PRJNA924553.

## Ethics statement

The animal study was reviewed and approved by Ethics Committee of Animal Experiments of Qingdao Agricultural University.

## Author contributions

PG, XL, and JY acquired experimental data. PG and JY drafted the manuscript. ZW, XW, XT, HS, and ZQ were involved in study design, study supervision, and obtaining funding. All authors contributed to the article and approved the submitted version.

## Funding

The study was supported by Shandong Provincial Natural Science Foundation (ZR2021MC173), Shandong Provincial Major Project of the New-Old Kinetic Energy Conversion: [2020] No. 1220. Horizontal project of Qingdao Agricultural University (661/1115010).

## Conflict of interest

Author TX was employed by QingDao Xnoba Biological Technology Co., Ltd.

The remaining authors declare that the research was conducted in the absence of any commercial or financial relationships that could be construed as a potential conflict of interest.

## Publisher’s note

All claims expressed in this article are solely those of the authors and do not necessarily represent those of their affiliated organizations, or those of the publisher, the editors and the reviewers. Any product that may be evaluated in this article, or claim that may be made by its manufacturer, is not guaranteed or endorsed by the publisher.
